# Clustering of patient-derived laboratory data: A privacy-preserving distributed approach to enhance clinical laboratory quality control and regional external quality assessment

**DOI:** 10.1371/journal.pone.0359083

**Published:** 2026-10-16

**Authors:** Chao Song, Qian Chen, Chunsheng Qu, Liqing Xu, Zhiming Shan, Jun Zhou, Weixing Li

**Affiliations:** 1 Laboratory Medicine Center, Zhejiang Center for Clinical Laboratories, Zhejiang Provincial People’s Hospital, Affiliated People’s Hospital, Hangzhou Medical College, Hangzhou, Zhejiang, China; 2 Department of Medical Affairs, Zhejiang Provincial People’s Hospital, Affiliated People’s Hospital, Hangzhou Medical College, Hangzhou, Zhejiang, China; 3 Department of Cilinical laboratory, Lishui Hospital of Wenzhou Medical University, The First Affiliated Hospital of Lishui University, Lishui People’s Hospital, Lishui, Zhejiang, China; 4 Department of Laboratory Medicine, Songyang People’s Hospital, Lishui, Zhejiang, China; 5 Department of Laboratory Medicine, Hangzhou Children’s Hospital, Hangzhou, Zhejiang, China; Kintampo Health Research Centre, GHANA

## Abstract

**Background:**

Traditional quality control (QC) has inherent limitations, and there are still various challenges existing in patient-based real-time QC (PBRTQC) and patient-based quality assurance (PBQA). We developed an integrated PBRTQC-PBQA model using density-based clustering and evaluated its effectiveness as a regional clinical laboratory quality assurance tool.

**Methods:**

A 20-month multicentre study across seven hospitals analysed five haematological analytes(white blood cell count [WBC], red blood cell count [RBC], haemoglobin [HGB], platelet count [PLT], and mean corpuscular volume [MCV]). Custom Clustering-PBRTQC-PBQA(CPP) models were developed using log/Box-Cox transformation, 3D spatial construction with companion analytes determined by distribution morphology, Mahalanobis distance filtering (chi-square = 0.3, ~ 47% exclusion rate), and iterative OPTICS clustering to identify hospital-specific core reference populations. A privacy-preserving distributed architecture transmitted only aggregated statistical summaries. Median/EWMA harts (PBRTQC) and Z-score/relative bias charts (PBQA) were created alongside a closed-loop quality improvement system and calibration event verification.

**Results:**

The CPP model reduced EWMA alert episodes by 25.0% (92–69) while increasing 1-3S alert episodes by 31.2% (64–84) compared with conventional moving-average PBRTQC, demonstrating selective elimination of systematic bias signals while preserving sensitivity to random errors (net change: 180–173 episodes, −3.9%). Overall calibration consistency was 82.3% (95% CI: 74.1%−88.7%). Among 52 confirmed true-positive alerts, 37 (71.2%) were detected without concurrent internal QC out-of-control signals, demonstrating the model’s capacity to capture low-magnitude biases that conventional IQC misses.

**Conclusion:**

Density-based clustering underpins the PBRTQC-PBQA model’s effectiveness in deriving hospital-specific core reference values, filtering unconfirmed QC alerts, validating calibration, and enhancing regional test consistency. The model’s ability to detect systematic biases not captured by IQC underscores its value as a complementary quality assurance layer. Its lightweight design supports regional deployment.

## Introduction

The accuracy of clinical laboratory test results is crucial for evidence-based medicine, with quality control (QC) ensuring their reliability. Currently, laboratories primarily use physical QC materials alongside internal quality control (IQC) to assess imprecision, and external quality assessment/proficiency testing (EQA/PT) to evaluate different methods [1–4]. However, traditional QC has notable limitations: matrix effects between QC materials and patient samples can distort error detection [5]; high costs restrict QC to periodic checks, delaying the identification of systematic errors [6]; and it overlooks pre- and post-analytical errors [7]. Crucially, IQC is optimally designed to detect random errors and large acute systematic errors; however, slow-onset systematic biases — such as those caused by gradual reagent deterioration, instrument drift, or progressive hardware wear — may remain below the IQC detection threshold for extended periods [8]. Patient-based real-time quality control (PBRTQC) has been proposed as a complementary approach to identify these low-magnitude, persistent systematic biases that conventional IQC characteristically misses. PBRTQC refers to longitudinal monitoring of a single laboratory’s analytical performance using that laboratory’s own patient data — functioning as a supplement to internal quality control (IQC). Patient-based quality assurance (PBQA) extends this concept to inter-laboratory comparison using patient data — functioning as a supplement to EQA/PT.

PBRTQC supplements traditional QC methods by utilising patient results and statistical algorithms [8–10]. However, it faces challenges in defining reference populations and managing data heterogeneity [11]. Hospital populations are heterogeneous, and simple outlier elimination fails to isolate stable reference groups, resulting in high false out-of-control rates [8,12]. PBQA further extends the application of patient-derived data from single-laboratory IQC to regional inter-laboratory EQA. This approach enables high-frequency, dynamic comparison of regional laboratory testing performance using real patient data, thereby addressing the limitations of traditional EQA, such as low sampling frequency and poor timeliness [13,14]. Nevertheless, the integrated application of PBRTQC and PBQA in regional laboratory quality assurance remains in the exploratory stage. There is a lack of practical pilot studies on how to achieve the deep integration of IQC and EQA through the combination of PBRTQC and PBQA, and thereby further improve the standardisation of regional laboratory testing.

To address these challenges and explore a more efficient regional laboratory quality assurance model tailored to clinical practice in China, we conducted a multicentre pilot study in a designated region. We developed an integrated model, Clustering-PBRTQC-PBQA (CPP), alongside a quality assessment framework to improve the quality of regional laboratory testing.

## Methods

### Data collction

This multi-centre cohort study was conducted over 20 months, from 01/03/2024 to 31/10/2025, involving seven hospitals in Zhejiang Province, China: one provincial grade A tertiary hospital (No. 6), two municipal grade A tertiary hospitals (Nos. 1101 and 11101), two district-level grade B tertiary hospitals (Nos. 1151 and 1285), and two county-level grade A secondary hospitals (Nos. 1147 and 11121). Data included all haematology test reports issued during the study, focusing on five analytes: white blood cell count (WBC), red blood cell count (RBC), haemoglobin (HGB), platelet count (PLT), and mean corpuscular volume (MCV). Report time, patient age, source, and report number were recorded, with all personal identifiers removed. Inclusion criteria were: (1) outpatient reports only, excluding emergency and fever clinics (emergency and fever clinic patients more frequently present with acute-phase haematological changes that would disproportionately populate outlier clusters; their exclusion yields a more stable physiological reference population); (2) reports issued between 7:30 am and 5:00 pm; (3) patients aged 14–80 years. Data used in this study were accessed for research purposes from 01/09/2025 to 31/12/2025. All data were obtained from outpatient participants and were used strictly for this research on clinical laboratory quality control and regional external quality assessment with appropriate ethical approval.

Regarding the clinical characterization of excluded samples: rather than relying on linked individual‑level diagnostic codes for pre‑filtering, this study retained all routine outpatient samples and implemented algorithm‑driven automatic removal of aberrant cases. This strategy avoids the complexity of manual pre‑screening for predefined disease subgroups such as patients with haematological disorders (e.g., leukaemia, myelodysplastic syndrome, aplastic anaemia), oncology patients receiving myelosuppressive chemotherapy, and individuals with chronic kidney disease — populations whose haematological profiles deviate markedly from physiological reference distributions. This algorithm‑centred exclusion approach is consistent with core principles of established PBRTQC methodology [11,12], which aim to stabilise the reference population by removing pathological‑origin outliers. Formal linkage to individual patient diagnostic codes would require additional IRB approval and data governance procedures outside the scope of the current ethical approval; such validation is reserved for future prospective implementation work.

### CPP models training for individual laboratories

(1) Log transformation. Models for each hospital were trained on 12 months of data (01/03/2024–30/11/2024). Log transformation was applied alongside robust scaling using the interquartile range to minimise outlier effects. For analytes not normalised by natural logarithm, a Box-Cox transformation was used to approximate normal distribution [15]. Laboratory data are typically right-skewed; transformation to approximate normality improves the performance of distance-based algorithms.
(2) Standardized 3D construction and data filtering. The preprocessed 3D data (one primary plus two companion analytes) were combined into a 3D matrix. The selection of companion analytes for 3D reference space construction was determined empirically by examining the distribution morphology of data points across all candidate analyte combinations. We systematically generated and visually inspected 3D scatter plots for every possible pairing of the five primary analytes (WBC, RBC, HGB, PLT, and MCV). The companion pairings were assigned as follows: WBC was paired with RBC and PLT; RBC with WBC and PLT; PLT with WBC and RBC; HGB with WBC and PLT; and MCV with WBC and PLT. This data-driven approach ensures that each 3D reference space possesses the spherical core morphology required for stable OPTICS cluster identification, rather than relying on a priori physiological assumptions that may not hold at the population level. As illustrated by Hospital 1101, 3D scatter plots of PLT‑WBC‑RBC companion pairings before and after Mahalanobis‑distance analysis showed distinct temporal performance patterns (Fig 1), demonstrating the existence of a hospital‑specific core reference population.

**Fig 1:**
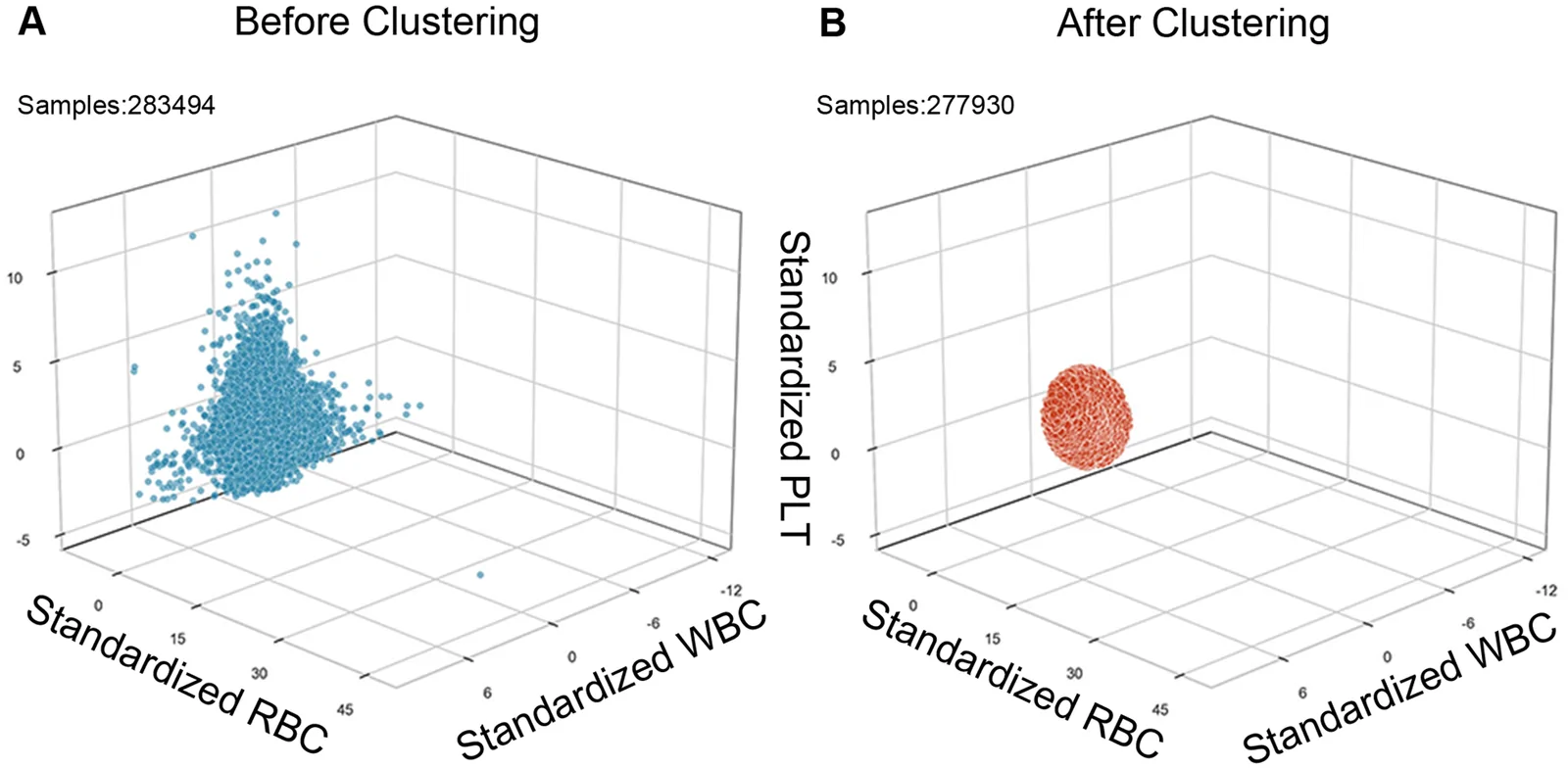
3D scatter plots of standardised clinical analytes before and after Mahalanobis-distance analysis. Panel A (Before Clustering) displays standardised values of three analytes (RBC, WBC, and PLT) from the original dataset, revealing a dispersed, right-skewed distribution with a loosely defined dense core and scattered high-value outliers. Panel B (After Clustering) shows the filtered core cluster (red), which is compact, with outliers removed.

(3) Iterative OPTICS density-based clustering. Iterative OPTICS clustering involved iterative OPTICS applied to standardised 3D data post-Mahalanobis distance(MD) filtering. OPTICS was selected over alternative clustering approaches because it can identify clusters of varying density without requiring a fixed neighbourhood radius, handles variable cluster densities critical for heterogeneous clinical populations, and enables iterative cluster extraction with statistical testing at each step. (a) initial clustering identified the largest dense cluster as the core reference population; (b) remaining outliers were re-clustered to find candidate groups for merging; (c) Hotelling’s T^2^ and Box’s M tests assessed multivariate similarity (merging if *P* > 0.05); (d) iterations continued until no further clusters could be merged. The final stable cluster was designated the core reference population; residual points were deemed pathological outliers. Key parameters: relative density threshold ξ = 0.01, minimum cluster size = 0.2, merging P = 0.05.
(4) Hyperparameter selection and sensitivity analysis. Key hyperparameters were empirically optimised on the dataset (01/03/2024–31/10/2025): (a) MD threshold (χ^2^ = 0.30): MD threshold of 0.3 was applied to identify the core population, yielding a mean sample exclusion rate of approximately 46% (range 41–50% across hospitals and analytes). This exclusion rate is consistent with the expected proportion of diagnostically abnormal samples in a tertiary hospital setting. By isolating the core (predominantly healthy) subpopulation more stringently, this design provides a rigorous test of whether the clustering step itself contributes to alert reduction, rather than relying on marginal sample exclusion. (b) To verify that the identified core population was not an artefact of a single parameter set, we performed a systematic sensitivity analysis. The MD threshold was held fixed, while the three OPTICS hyperparameters were varied independently and in combination: the steepness parameter ξ ∈ {0.005, 0.01, 0.02, 0.03, 0.05}, the neighbourhood size min_samples ∈ {10, 15, 20, 25, 30}, and the minimum cluster size min_cluster_size ∈ {0.10, 0.15, 0.20, 0.25, 0.30}. For each hospital, analyte, and parameter combination, the iterative OPTICS procedure was rerun, and the resulting core-cluster membership was compared with that obtained under the baseline parameters (ξ = 0.01, min_samples = 20, min_cluster_size = 0.20) using the Jaccard similarity coefficient, J = |A ∩ B| / |A ∪ B|, where A and B denote the sets of core-cluster sample indices. A Jaccard value close to 1.0 indicates that the core population is essentially invariant to that parameter perturbation. Due to the substantial computational burden of the full parameter grid (5 × 5 × 5 = 125 combinations per hospital per analyte, applied to datasets exceeding 60,000 samples per hospital), the sensitivity analysis was performed on a representative three-month period (01/09/2025–31/10/2025) across all seven participating hospitals. This period encompassed typical daily sample volumes and was free of known major calibration interventions, ensuring that the distribution characteristics were representative of routine operations. The heatmap summarising the ξ × min_cluster_size landscape (at min_samples = 20) was shown in S1 Fig.

This approach allows development of a hospital-specific CPP model with customised core reference population selection. Fig 2. illustrates the single-hospital data processing flow, outlining the process from raw data acquisition to QC implementation.

**Fig 2:**
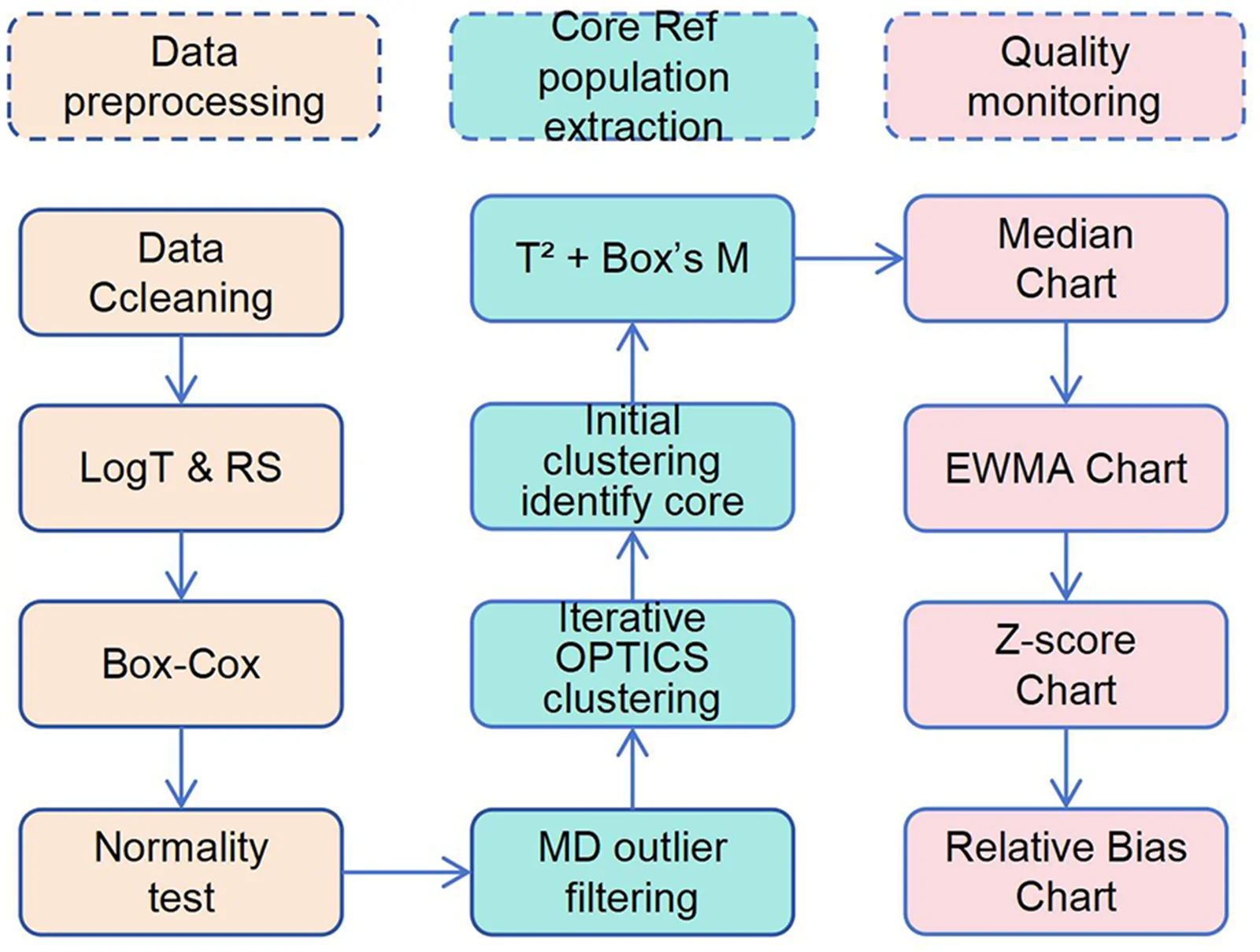
Flow chart of data processing and quality control analysis for a single hospital. The workflow consists of three sequential stages, shown by arrows: data preprocessing (yellow) for normalising haematological analytes; clustering and localisation (green) to identify stable physiological populations; and quality control analysis (red) using four-chart multidimensional monitoring. Abbreviations: LogT & RS, log transformation and robust scaling; MD, Mahalanobis Distance; T^2^ + Box’s M, Hotelling’s T^2^ and Box’s M test for merging non-significant clusters.

### Quality control charts of Clustering-PBRTQC-PBQA

The Harrell-Davis median (HD50) represented the core reference population’s statistical indicator. For PBRTQC (as IQC), longitudinal quality control charts—median and EWMA—were created for each laboratory. For PBQA (as regional EQA), horizontal comparison charts—Z-score and relative bias—were developed for cross-institutional comparison.

Median charts: Daily HD50 values were plotted on Levey-Jennings control charts, using the mean and standard deviation (SD) as control limits for quality monitoring. Classic Westgard rules (1_3S_, 2_2S_) were applied to detect abnormalities [10].

EWMA chart updates weighted averages daily, plotted as control charts. Daily EWMA results are calculated using the formula: *EWMA_day* = λ × *x_day* + (1-λ)×*EWMA_(day-1)*. *x_day* represents the daily core population characteristic value, and λ is the smoothing parameter (0 < λ ≤ 1). This study used a fixed λ of 0.1 [16]. Alerts were triggered when preset control limits were exceeded [17,18], indicating that the hospital’s cumulative systematic errors had reached a significant level. Control limits were calculated using *target_block_mean*±3 × SD/sqrt(*block_size*). The *target_block_mean* and *SD* refer to the mean and standard deviation of the rule-setting interval, with *block_size* set to seven days (one week).

Z-score chart displays daily Z-scores for each hospital relative to the regional mean of characteristic values from seven hospitals. It clearly shows the extent and direction of each hospital’s deviation from the regional average. Alerts were triggered when Z-scores consistently exceeded ±2, signalling potential significant systematic errors within the hospital.

Relative bias chart: This chart shows the percentage of relative bias for each hospital’s daily characteristic values compared to the regional mean. It quantifies bias to help establish acceptable performance standards.

### Privacy-preserving distributed analysis and real-time linkage mechanism

A privacy-preserving distributed analysis architecture was implemented to ensure the completeness and security of patient data reports, reduce the risk of privacy breaches, and standardise nomenclature across laboratory information systems (LIS) [19,20]. Customised CPP models were embedded in each hospital’s LIS, with only computed statistical summaries uploaded to the regional evaluation centre. Notably, this architecture differs from classical federated learning in that no model parameters or gradients are shared among participating sites. Each hospital independently trains its local clustering model, and only aggregated statistical summaries (daily HD50, EWMA values per analyte) are transmitted to the central platform. Privacy is preserved through data minimisation: no patient-level data leaves each hospital’s LIS. This approach follows the “minimal data sharing” principle of privacy-by-design.

Since 01/12/2024, a standardized quality improvement mechanism based on CPP has been implemented. When a laboratory’s PBRTQC triggers an abnormal early alert, the regional evaluation platform promptly reviews the laboratory’s PBQA horizontal comparison data to determine whether the abnormal bias is a laboratory-specific systemic error or a common error affecting multiple regional laboratories. Upon alert triggering, the investigation protocol included: (1) review of IQC results for the preceding 72 hours; (2) instrument maintenance log review; (3) reagent/calibrator lot change verification; (4) review of pre-analytical conditions (specimen collection, transport); and (5) if no cause was identified within 48 hours, classification as an unconfirmed alert. This allowed retrospective evaluation of the relationship between PBRTQC alerts and concurrent IQC performance. PBRTQC and IQC are designed to be complementary, not interchangeable: IQC uses high-concentration control materials to detect acute, large-magnitude errors, while PBRTQC using the EWMA chart is designed to accumulate evidence for slow-onset, low-amplitude systematic biases that may not exceed IQC warning limits within a single day [8,9]. Requiring IQC concordance as a precondition for PBRTQC alert validation would therefore systematically underestimate the model’s true clinical value.

### Verification of CPP against real calibration events

Calibrations were conducted for five analytes across seven hospitals during the study period. Calibration factors for analytes requiring correction were recorded as the ratio of the new calibration coefficient to the original calibration coefficient. Data surrounding each calibration event were divided into two groups: a pre-calibration period (14 days, encompassing the calibration day and the 13 preceding days) and a post-calibration period (14 days following calibration). The mean values of the five analytes for these two periods were calculated, and an independent-samples t-test was applied to assess whether the difference between the means was statistically significant (P < 0.05). Calibration consistency was defined as the concordance between the direction of the calibration factor (factor > 1 indicating expected increase, < 1 indicating expected decrease) and the observed post-calibration directional change in analyte values. Consistency was categorised as: (i) statistically significant and directionally concordant (Sig & Cons, P < 0.05); (ii) directionally concordant but not statistically significant (Cons, NS, P ≥ 0.05); and (iii) directionally discordant (Incons).

### Head-to-head comparison with conventional PBRTQC

To evaluate whether the observed differences in alert performance were attributable to the clustering step itself rather than to more restrictive sample filtering, we compared the CPP method against a conventional PBRTQC baseline on the identical dataset. To ensure a fair and isolated comparison, both methods shared: (i) the same daily statistic—the Harrell–Davis median (HD50); (ii) the same fixed reference period (01/12/2024 to 31/10/2025); and (iii) the same control rules. The sole methodological difference was the sample basis for each daily HD50: the conventional method used all valid patient samples collected that day, whereas the CPP method used only samples assigned to the core cluster after Mahalanobis distance filtering and iterative OPTICS clustering.

### Ethics statement

This retrospective study analysed existing anonymised patient laboratory data for evaluation of detection system bias and laboratory quality control. The observation object was the clinical detection system rather than individual patients. No physical intervention, treatment, or behavioral manipulation was performed on any enrolled patients. All personal identifiable patient information was strictly anonymized and desensitized to ensure complete protection of patient privacy and data confidentiality. This study was approved by the Ethics Committee of Zhejiang Provincial People’s Hospital (Approval No. ZJPPHEC 2025I(249)). Owing to its retrospective nature and minimal risk to participants, a waiver of informed consent was granted by the ethics committee.

## Results

### Filtering of abnormal data by the CPP models

The CPP models excluded a mean of 46.9% of daily samples (range 40.8–50.3%) through MD filtering (χ^2^ = 0.30), retaining a homogeneous core cluster for HD50 calculation. Retrospective analysis of the excluded outliers revealed their department origin: haematology department 41.2%, oncology 23.8%, nephrology 11.5%, and other departments 23.5%. This distribution is consistent with expected sources of pathological haematological abnormalities. The core reference population and excluded samples showed no statistically significant difference in age distribution (Kolmogorov-Smirnov test, P > 0.05 for all seven hospitals). Core HD50 values remained consistent (mean deviation < 0.5%) regardless of outlier inclusion on 10 randomly selected days per hospital.

### Verification of CPP alerts

During the historical data filtering and model training phase (01/03/2024–30/11/2024), each laboratory successfully developed customised CPP models for five analytes. Since the launch of the early alert feedback mechanism in December 2024, the head-to-head comparative analysis covering seven hospitals and five analytes, with a median monitoring duration of 335 days (range, 330–335 days). CPP models incorporating median and EWMA charts produced a total of 173 quality alerts. After integrated PBQA evaluation and on‑site laboratory verification, 30.1% (52/173) of all alerts were validated as true positives corresponding to genuine systemic analytical bias. Median time-to-detection also improved (3.1 days, IQR: 2–5 vs. 4.2 days, IQR: 3–7). Analysis of the relationship between the 52 confirmed true-positive PBRTQC alerts and concurrent IQC status revealed a clinically important finding: of the 52 confirmed true-positive alerts, 15 (28.85%) were accompanied by concurrent IQC out-of-control events within the same 72-hour window. However, 37 (71.15%) confirmed true-positive PBRTQC alerts occurred in the absence of concurrent IQC out-of-control signals — that was, these systematic biases were detected by the CPP model but would have been missed by IQC alone during the same monitoring period.

Overall, relative to conventional PBRTQC (180 total alert episodes), the CPP model reduced total alert episodes to 173, representing a 3.8% reduction and a decline in monthly alert frequency from 0.46 to 0.45 alerts per analyte-month. The reduction was concentrated in EWMA alerts, which decreased by 25.0% (from 92 to 69 episodes), while 1_3S_ alerts increased by 31.3% (from 64 to 84) and 2_2S_ alerts decreased by 16.6% (from 24 to 20). The pronounced EWMA reduction was the most clinically relevant finding: EWMA charts detect systematic analytical shifts—the error type most likely to require instrument intervention—whereas 1_3S_ alerts frequently represent random, transient excursions. By restricting the HD50 calculation to a homogeneous core subpopulation, the Clustering-enhanced approach reduced the daily median’s susceptibility to patient-mix fluctuations, thereby suppressing EWMA signals driven by changes in case composition rather than by genuine analytical drift.(S1 Table, part A)

The alert reduction was analyte-dependent and consistent with the method’s theoretical rationale (S1 Table, part B). EWMA alerts were eliminated entirely for WBC (from 7 to 0 episodes) and reduced by 72.2% for PLT (from 18 to 5)—the two analytes with the greatest inter-patient biological variability. RBC and HGB showed intermediate EWMA reductions (20.0% and 8.6%, respectively). In contrast, MCV showed EWMA a net increase in total alerts (from 19 to 23, + 21.1%), driven by a 120% increase in 1_3S_ episodes. This pattern is expected: because MCV exhibits minimal inter-patient variability, the core cluster closely resembles the overall patient population, and the tighter control limits (narrower ±3SD boundaries from the reduced core-cluster variance) increase 1_3S_ sensitivity without a commensurate reduction in false EWMA signals.

At the individual hospital level, alert reductions were observed for four of seven hospitals (Hospital 6: −30.0%, Hospital 1151: −25.9%, Hospital 1285: −25.0%, Hospital 1101: −20.0%), while three hospitals showed net increases (Hospital 1147: + 30.8%, Hospital 11101: + 29.6%, Hospital 11121: + 8.3%). The increases at Hospital 1147 and Hospital 11121—the hospitals with the smaller sample volume (29,242 and 38,698 samples, respectively)—were concentrated in EWMA alerts for RBC, HGB, and MCV, likely reflecting reduced core-cluster stability at lower sample sizes.

### Case 1: System bias alerts due to instrument replacement

Monitoring results after updating the analytical instrument at Hospital 1285 (MCV) are shown in Fig 3. From March to late April 2024, a marked negative analytical bias appeared. The PBRTQC Median chart showed a sharp drop in HD50 with multiple breaches of ±3 SD control limits, while the EWMA chart triggered consecutive multi-day alerts below the threshold. The PBQA relative bias consistently exceeded −3%. After targeted corrective actions, MCV values gradually recovered by May 2025. However, from May 2025, Hospital 1285 began using a different brand of analytical instrument in the new laboratory. Between May and mid-July 2025, the PBRTQC showed an abrupt negative shift in both the Median and EWMA charts, with values below lower control limits. Simultaneously, the PBQA Z-score and relative bias charts were significantly lower than peer group values. Recalibration in mid-July corrected the systematic bias.

**Fig 3:**
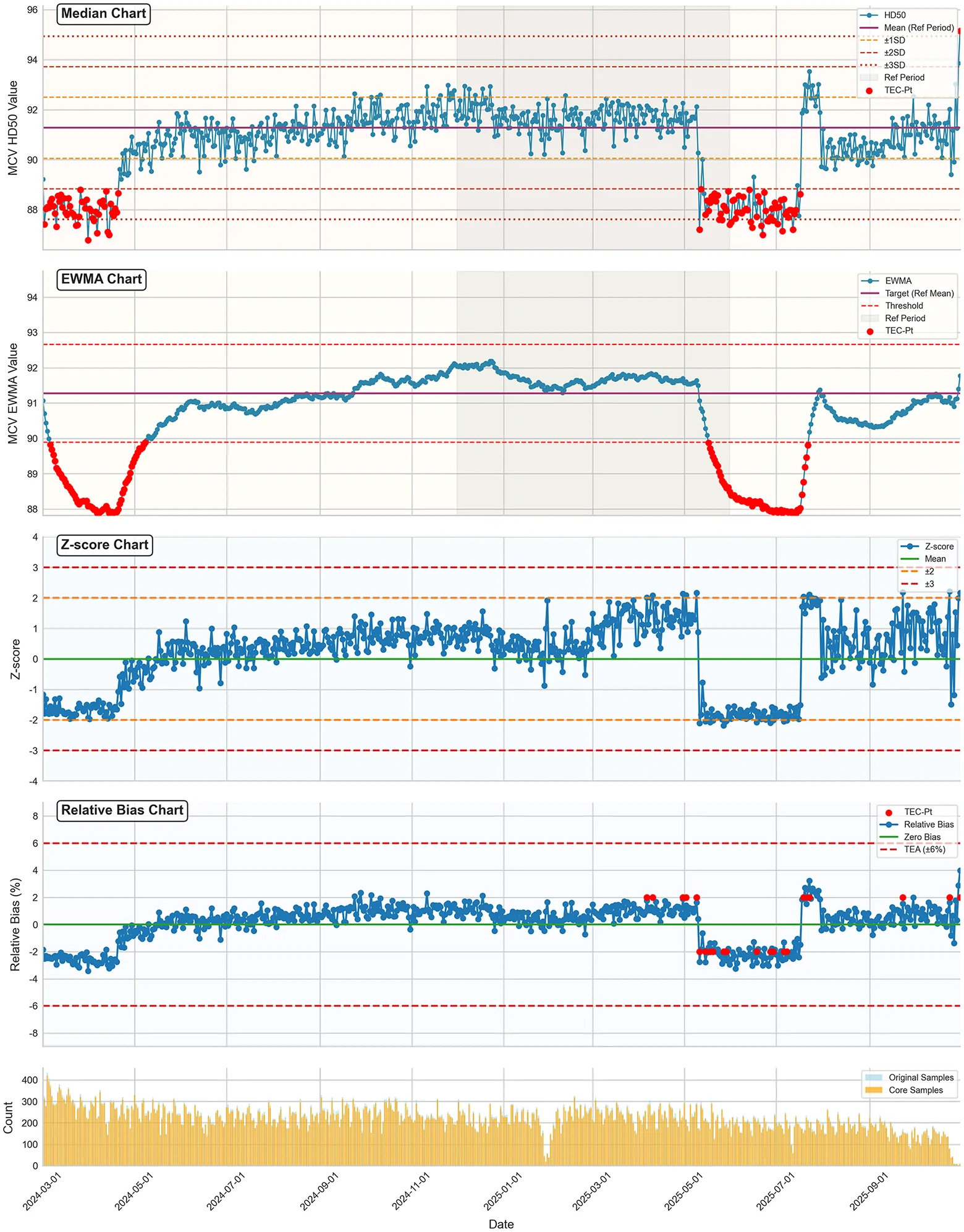
The Clustering-PBRTQC-PBQA charts for MCV at Hospital 1285 (01/03/2024–31/10/2025). Blue dots indicate values within control limits, red dots denote anomalies, and the grey shaded area represents the baseline reference interval. The figure comprises five components: a median chart (top panel) of daily Harrell-Davis median values; an EWMA chart (second panel) reflecting gradual, systematic trends; a Z-score chart (third panel) relative to seven hospitals; a relative bias chart (fourth panel) against the mean of the seven hospitals; and a daily test volume chart (bottom panel) with yellow bars representing retained results and blue bars indicating excluded results. A cluster of out-of-control points appearing in May 2025 (red dots in EWMA chart) following instrument replacement at Hospital 1285 indicates a systematic downward bias, subsequently corrected by recalibration in mid-July 2025 (red arrow). The Z-score chart confirms that this bias was hospital-specific.

### Case 2: Early alerts of instrument abnormalities

In mid-August 2025, an early systematic negative bias was detected with simultaneous alerts for RBC (Fig 4) and WBC (Fig 5) at Hospital 1151. For RBC, the median chart HD50 dropped, EWMA triggered consecutive alerts below the threshold, the Z-score fell below −3, and relative bias neared the TEa limit of −6%. For WBC, the median chart HD50 also fell to lower control limits, EWMA triggered alerts below the threshold, and relative bias exceeded −20%, well beyond the TEa limit of 15%. On-site laboratory investigation identified the cause as leakage in the instrument’s sample aspiration line. A gradual local leak worsened to a severe pipeline failure by mid-to-late August 2025, causing temporary instrument shutdown and maintenance. After targeted corrective actions, both analytes rapidly recovered.

**Fig 4:**
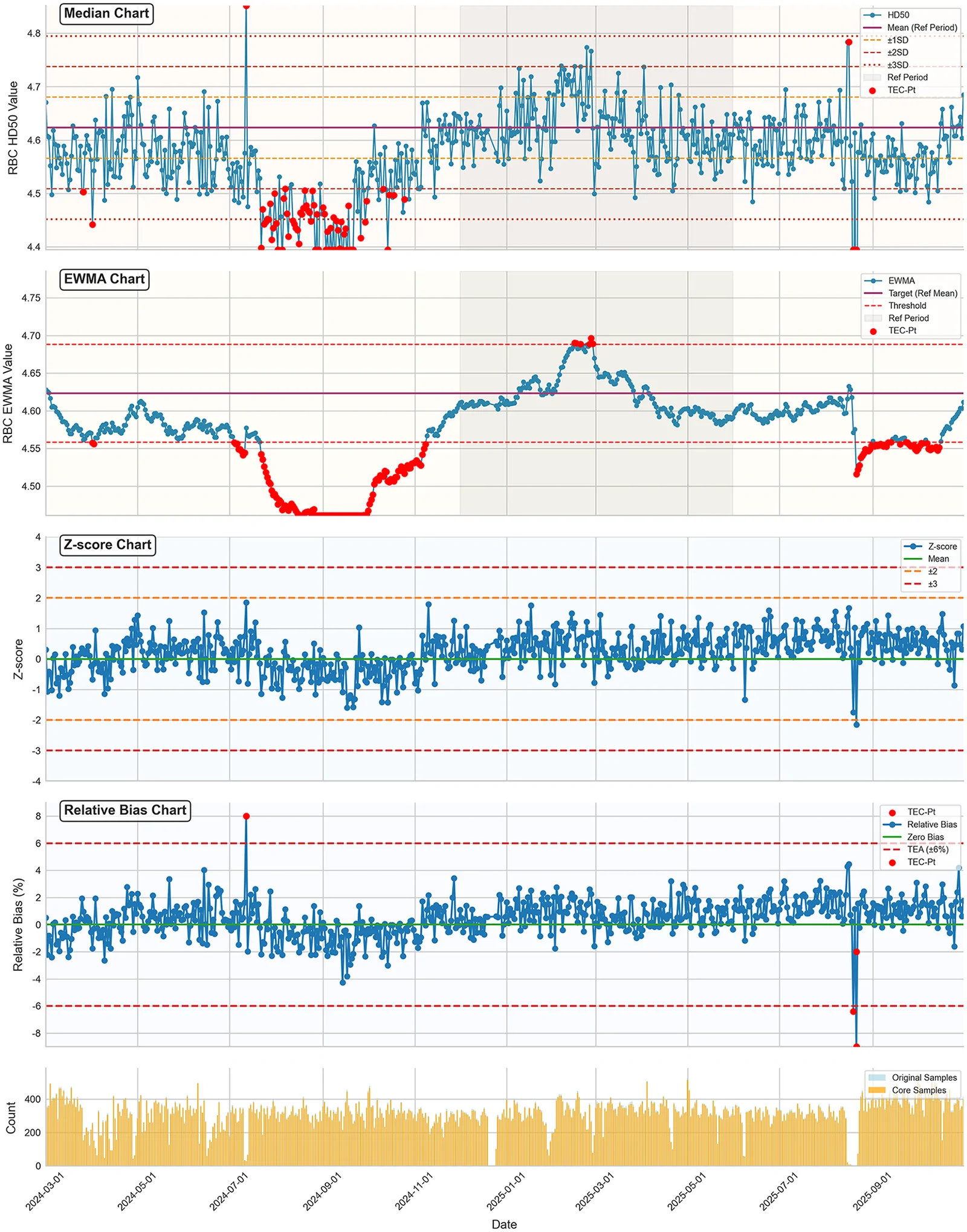
RBC Clustering-PBRTQC-PBQA monitoring charts at Hospital 1151 (01/03/2024–31/10/2025).

**Fig 5:**
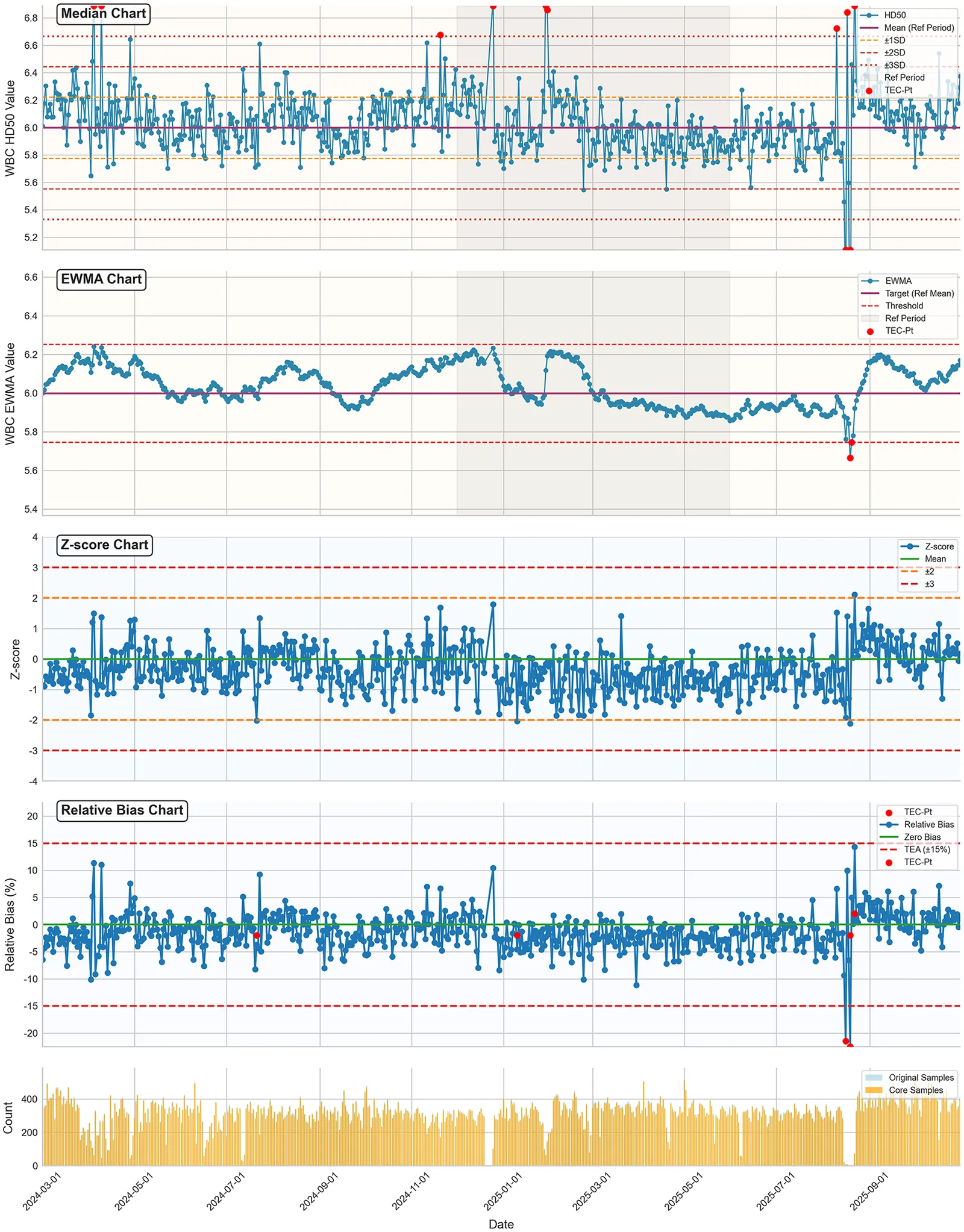
WBC Clustering-PBRTQC-PBQA monitoring charts at Hospital 1151(01/03/2024–31/10/2025). Blue dots indicate values within control limits, red dots denote anomalies, and the grey shaded area represents the baseline reference interval. Concurrent WBC and RBC alerts (mid-August 2025) pointed to a shared pre-analytical cause (aspiration line leak), distinguishable from reagent-specific errors.

Blue dots indicate values within control limits, red dots denote anomalies, and the grey shaded area represents the baseline reference interval. A systematic negative deviation beginning mid-August 2025 (red dots) reflects progressive aspiration line leakage, which is hospital-specific (confirmed by Z-score chart falling below −3).

### Verification to the real calibration events

Calibration effectiveness across seven hospitals (Table 1) showed an overall 82.3% (95% CI: 74.1%–88.7%) consistency between theoretical calibration factor trends and post-calibration analyte changes: 38.7% were statistically significant (Sig & Cons, P < 0.05), 43.6% consistent but non-significant (Cons, NS), and 17.7% inconsistent (Incons). Consistency by analyte was as follows: RBC 80.0% (12/15), MCV 85.7% (12/14), HGB 63.6% (7/11), WBC 83.3% (10/12), and PLT 90.0% (9/10). The observed 82.3% consistency is significantly higher than the 50% expected under a null-hypothesis scenario of random directional concordance (binomial test, P < 0.001). Inter-hospital agreement in core reference HD50 values was excellent across all analytes (intra-class correlation coefficients: WBC 0.91, RBC 0.96, HGB 0.89, PLT 0.93, MCV 0.94). Regarding the 17.7% directionally discordant events: these are not “false positives” of the calibration verification system, but rather cases where the expected post-calibration shift was not detectable in the patient-data signal. Root-cause analysis of these 11 discordant events (out of 62 total calibration events) indicated: 5 cases with calibration factor magnitude < 1.005 (sub-threshold shifts); 4 cases with possible superimposed pre-analytical variation during the post-calibration window; and 2 cases interpreted as incomplete calibration correction, which were subsequently reported back to the respective hospitals for further investigation. This analysis underscores the diagnostic value of the CPP calibration verification module.

**Table 1:** Calibration events and consistency assessment.

Hospital ID	Date	Analyte	Calibration Factor	*p*-value	Consistency Assessment	Hospital ID	Date	Analyte	Calibration Factor	*p*-value	Consistency Assessment
6	02/09/2024	MCV	1.012	0.459	Incons	1101	13/03/2024	HGB	1.024	0.153	Incons
6	02/09/2024	PLT	1.049	0.172	Cons,Ns	1101	13/03/2024	MCV	1.020	0.001	Sig& Cons
6	02/09/2024	RBC	0.982	0.331	Cons,Ns	1101	13/03/2024	PLT	0.951	0.037	Sig& Cons
6	02/09/2024	WBC	1.023	0.244	Cons,NS	1101	13/03/2024	RBC	0.985	0.264	Cons,NS
6	26/02/2025	MCV	1.002	0.000	Sig& Cons	1101	13/03/2024	WBC	1.023	0.223	Cons,NS
6	26/02/2025	PLT	1.042	0.008	Sig& Cons	1101	11/09/2024	HGB	1.019	0.013	Sig& Cons
6	26/02/2025	WBC	0.958	0.000	Sig& Cons	1101	11/09/2024	MCV	0.986	0.009	Sig& Cons
1285	10/05/2025	HGB	1.023	0.123	Cons,NS	1101	11/09/2024	PLT	0.968	0.032	Sig& Cons
1285	10/05/2025	MCV	0.947	0.000	Sig& Cons	1101	11/09/2024	RBC	1.026	0.000	Sig& Cons
1285	10/05/2025	PLT	0.962	0.479	Cons,NS	1101	10/03/2025	MCV	1.026	0.171	Cons,NS
1285	10/05/2025	RBC	1.035	0.000	Sig& Cons	1101	10/03/2025	PLT	1.054	0.025	Sig& Cons
1285	21/05/2024	RBC	1.010	0.024	Incons	1101	10/03/2025	RBC	0.974	0.294	Cons,NS
1285	21/05/2024	HGB	1.026	0.130	Incons	1101	10/03/2025	WBC	1.045	0.193	Incons
1151	15/03/2024	HGB	0.984	0.001	Sig& Cons	1101	27/08/2025	HGB	0.982	0.832	Cons,NS
1151	15/03/2024	MCV	1.011	0.491	Incons	1101	27/08/2025	MCV	1.012	0.000	Sig& Cons
1151	15/03/2024	PLT	0.989	0.302	Incons	1101	27/08/2025	PLT	1.052	0.000	Sig& Cons
1151	15/03/2024	RBC	0.989	0.292	Cons,NS	1101	27/08/2025	RBC	1.012	0.090	Cons,NS
1151	15/03/2024	WBC	1.024	0.912	Incons	1101	27/08/2025	WBC	0.941	0.285	Cons,NS
1151	21/09/2024	MCV	0.993	0.403	Cons,NS	11101	30/07/2024	WBC	0.984	0.499	Cons,NS
1151	21/09/2024	RBC	1.017	0.000	Sig& Cons	11101	30/07/2024	MCV	1.019	0.003	Sig& Cons
1151	26/02/2025	HGB	0.985	0.007	Sig& Cons	11101	25/08/2025	HGB	1.026	0.589	Incons
1151	26/02/2025	MCV	0.990	0.005	Sig& Cons	11101	25/08/2025	RBC	1.024	0.027	Incons
1151	26/02/2025	PLT	1.051	0.748	Cons,NS	1147	26/04/2024	WBC	0.983	0.388	Cons,NS
1151	26/02/2025	RBC	0.987	0.0036	Sig& Cons	1147	26/04/2024	RBC	0.976	0.6912	Cons,NS
1151	26/02/2025	WBC	0.984	0.0192	Sig& Cons	1147	26/04/2024	MCV	1.023	0	Sig& Cons
1151	16/06/2025	HGB	1.026	0.2386	Cons,NS	1147	26/04/2024	HGB	1.01	0.8347	Incons
1151	16/06/2025	MCV	1.01	0.8502	Cons,NS	1147	01/11/2024	MCV	0.989	0.5246	Cons,NS
1151	16/06/2025	WBC	1.022	0.6667	Cons,NS	1147	01/11/2024	RBC	1.011	0.1475	Cons,NS
11121	27/08/2025	WBC	0.973	0.3649	Cons,NS	1147	16/04/2025	WBC	0.98	0.9908	Cons,NS
11121	27/08/2025	RBC	0.99	0.3711	Cons,NS	1147	16/04/2025	RBC	0.98	0.0018	Sig& Cons
11121	27/08/2025	PLT	1.018	0.0809	Cons,NS	1147	16/04/2025	HGB	0.982	0.0028	Sig& Cons

Caption: This table summarises the statistical validation outcomes of calibration events for five analytes across seven hospitals from 01/03/2024 to 31/08/2025. The primary evaluation indices include the calibration factor, the p-value from the independent samples t-test, and the consistency assessment of the calibration event and trend change. A calibration factor greater than 1 indicates a theoretically expected increase in post-calibration test results, whereas a factor less than 1 indicates a theoretically expected decrease. A p-value of less than 0.05 was considered statistically significant for differences in mean values before and after calibration. Consistency assessment was categorised into three levels: Sig & Cons (indicating a statistically significant difference in mean values after calibration [P < 0.05] and a change trend consistent with the theoretical trend of the calibration factor), Cons, NS (indicating a consistent change trend with the theoretical trend but no statistically significant difference in mean values [P ≥ 0.05]), and Incons (indicating an inconsistent change trend with the theoretical trend of the calibration factor)

Hospital 1101’s EWMA charts for CPP (Fig 6) showed 88.9% (16/18) calibration-trend consistency. After the 26 April 2024 calibration, RBC/HGB exhibited sustained downward deviations and MCV upward deviations, indicating suboptimal calibration, which normalised following recalibration on 1 November 2024.

**Fig 6:**
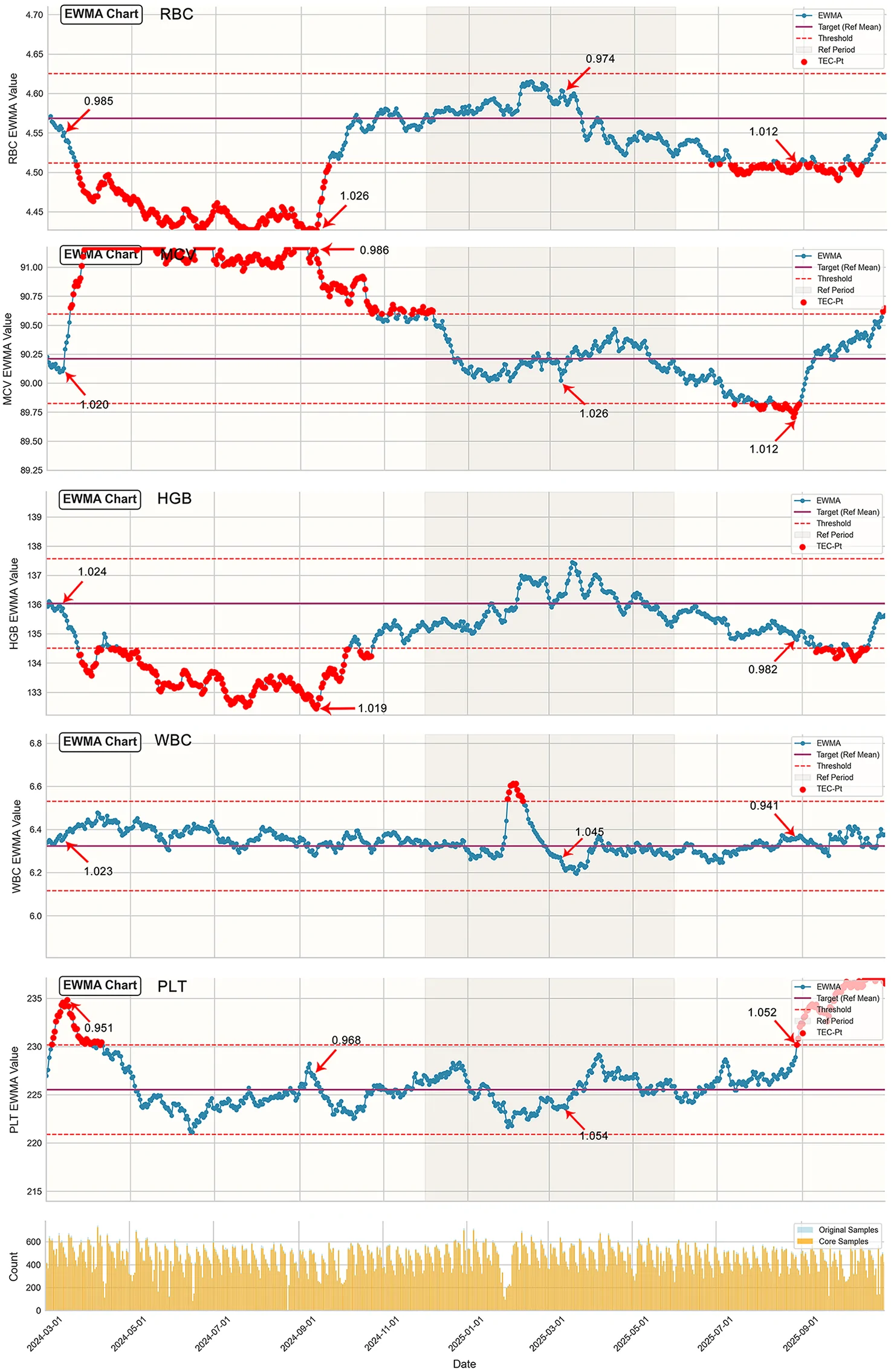
EWMA charts for five analytes at Hospital 1101 (Clustering-PBRTQC-PBQA monitoring, 01/03/2024–31/10/2025). The April 2024 calibration was followed by sustained downward deviations in RBC and HGB (red dots below lower control limits) and upward deviation in MCV, indicating suboptimal initial calibration. Successful normalisation following recalibration on 01/11/2024 (red arrow) illustrates the model’s utility in identifying inadequate calibration responses. Blue dots represent values within predefined control limits. The grey shaded area denotes the reference interval. Red arrows mark calibration events. The bottom panel shows daily valid test result counts (yellow bars).

## Discussion

This study used a lightweight density-based OPTICS clustering algorithm to develop CPP filtering models for individual laboratories. This method addressed ambiguous reference population definitions and high data heterogeneity in PBRTQC applications, while reducing implementation challenges in primary hospitals with limited hardware. It allows unified QC processes across laboratories with diverse information systems, without adding computational load to local LIS. In PBQA, the privacy-preserving distributed analysis architecture enables “contactless” sharing of regional quality control data, ensuring patient privacy and resolving the conflict between data sharing and privacy in regional medical quality control systems. This lightweight design suits rapid, large-scale regional deployment, requiring no complex model tuning or parameter adjustments for different sites.

The CPP system developed here uses real patient data, combining single-laboratory longitudinal PBRTQC monitoring with regional horizontal PBQA comparison. PBRTQC employs a hospital-specific core reference population to enable high-frequency daily IQC for individual laboratories [21]. PBQA expands patient data-based QC from single labs to entire regions, enabling daily EQA among regional laboratories. This approach overcomes the limitations of traditional periodic EQA by providing daily quality coverage and reducing unconfirmed alerts through regional comparisons. Traditional EQA frequency is limited by physical QC materials, transport costs, and value assignment, typically allowing only 2–4 assessments annually with limited lab participation, making weekly or daily evaluations impractical [22]. In contrast, CPP uses routine clinical patient data, eliminating issues related to matrix effects, sourcing, transport, and value assignment of physical QC materials, thus significantly cutting EQA costs [23]. By assessing inter-laboratory systematic biases via patient results, it enhances consistency of test outcomes across hospitals in a region.

The head-to-head comparison directly addresses whether the improvement in quality monitoring arises from the clustering step itself or from more restrictive sample filtering. By design, both methods used the same Harrell–Davis median, the same reference period, and the same Westgard and EWMA rules; the only variable was the daily sample basis. The 25.0% reduction in EWMA alert episodes demonstrates that the clustering step removes diagnostically heterogeneous sub-populations that would otherwise inflate the daily median’s variance and generate false systematic-error signals. This finding is consistent with the theoretical rationale for core-cluster identification: when the daily patient mix shifts toward pathological cases, the conventional all-patient median is pulled away from its baseline, triggering EWMA violations that reflect changes in case mix rather than analytical error. The Clustering-enhanced median, computed from the stable core subpopulation, is resistant to this confounding.

The concurrent 31.3% increase in 1_3S_ alert episodes warrants discussion. This increase reflects the narrower control limits (±3SD) that result from the reduced variance of the core-cluster-based reference: when the reference SD shrinks, a given absolute deviation is more likely to exceed 3SD. In PBRTQC practice, 1_3S_ alerts are generally considered less actionable than EWMA alerts, as they often represent random, self-correcting excursions rather than sustained shifts requiring intervention. The net effect represents a shift toward a more precise alert profile: fewer false systematic-error alarms, at the cost of modestly increased sensitivity to discrete outlier events. Whether this trade-off is favourable depends on the laboratory’s operational priorities; for laboratories prioritising the detection of genuine analytical drift while minimising alarm fatigue from patient-mix-driven EWMA signals, the Clustering-enhanced approach offers a clear advantage.

Calibration is essential in clinical laboratory quality management, correcting analytical bias and aligning test results with reference standards. Traditional post-calibration verification often depends on limited physical quality control materials, which are prone to matrix effects and may not accurately represent patient sample detection. The CPP method assesses calibration effectiveness using real patient data. The 82.3% calibration consistency rate reflects the model’s performance as a patient-data-based calibration verification tool, not as a replacement for existing post-calibration verification procedures. Rather than viewing the 17.7% non-concordance as a limitation, we interpret it as clinically meaningful signal: the 2 cases of detected incomplete calibration would not have been identified through physical QC material verification alone, demonstrating added value. The 9 cases of sub-threshold inconsistency appropriately reflect the inherent noise floor of any patient-data-based monitoring system, and are expected for calibration factors near the 1.000 identity value.

Generalizability. The model’s applicability to different settings warrants consideration. (a) Smaller laboratories: Daily volumes below 20–30 would compromise HD50 reliability and OPTICS cluster stability. Future development includes a pooled reference population approach and a floating block size adaptive algorithm. (b) Different geographic regions: The model derives hospital-specific reference populations, making it fundamentally generalisable. Validation in other provinces is planned. (c) Other test categories: Application to clinical chemistry is feasible with re-tuning. For microbiology and molecular testing, fundamentally different approaches are required.

### Limitation

This study faces several key challenges requiring attention in future optimisation of the CPP model. Firstly, as a pilot exploration, the central platform used a non-mandatory alert system: relevant staff were only notified of QC abnormalities without enforced corrective actions. Thus, bias correction depended on voluntary laboratory responses, explaining persistent systemic biases during validation. Future implementation will integrate alerts into routine quality management to prompt timely corrections, expected to reduce persistent bias unconfirmed alerts from the EWMA chart. Secondly, daily test volumes significantly affect PBRTQC robustness; performance correlates positively with testing counts. In primary hospitals with low daily volumes, the method is prone to fluctuations, undermining reliability. Thirdly, the current study did not link individual diagnostic codes to excluded samples. Although such linkage could directly verify the abnormal multivariate distribution profiles of excluded patients and quantify the accuracy of our sample exclusion strategy, the ultimate objective of this work is to eliminate reliance on diagnostic data. By avoiding diagnostic code linkage, the clustering algorithm achieves full automation and lightweight deployment, facilitating rapid real-world popularization of the model. Fourthly, the comparative analysis against conventional PBRTQC was performed retrospectively using the same dataset, rather than as a prospective head-to-head trial; prospective parallel comparisons incorporating standardised error injection protocols would provide higher-quality comparative evidence. Fifthly, the IQC-PBRTQC concordance analysis relied on investigation records from the clinical phase only, and is subject to the completeness and consistency of documentation practices across seven hospitals. CPP results reflect cumulative bias from pre-analytical (e.g., specimen handling) [24], analytical (e.g., instrument issues), and post-analytical (e.g., internal correction factors) sources [25–27]. Since data derive from patient outcomes, pinpointing error sources is not possible, necessitating thorough root cause analyses upon alerts. Future research will optimise intervention protocols, develop algorithms suited to sample sizes, and broaden monitoring to enhance the model’s reliability as a regional quality assurance tool.

## Supporting information

S1 Fig Heatmaps of Jaccard similarity for OPTICS parameter sensitivity analysis. Heatmaps show Jaccard similarity between core cluster membership derived from perturbed OPTICS parameters and the baseline setting (ξ = 0.01, min_samples = 20, min_cluster_size = 0.20). Each subplot corresponds to one hematology analyte (WBC, RBC, HGB, PLT, MCV). The color gradient indicates Jaccard similarity values, where values close to 1.0 denote stable core clusters. The blue box marks the baseline parameter combination. Sensitivity analysis was performed on a representative 3-month dataset from seven participating hospitals.
(TIF)

S1 Table Head-to-head comparison of alert episodes: Conventional PBRTQC vs. Clustering-PBRTQC-PBQA(CPP). Both methods adopted the same daily HD50 statistic, reference period (01/12/2024–31/10/2025), and control rules (Westgard 1_3S_, 2_2S_, and EWMA with λ = 0.1). The key distinction lies in the sample set used for daily HD50 calculation: conventional PBRTQC uses all valid patient samples, whereas CPP uses core-cluster samples obtained after MD filtering and iterative OPTICS clustering. Alerts are counted as episodes: consecutive days triggering the same rule are merged into one episode, and overlapping rule triggers are counted once. **Part A** summarizes overall alert episodes stratified by Westgard rule across all analytes and seven participating hospitals. **Part B** presents percentage changes in EWMA-derived alert episodes for individual hematology analytes.
(DOCX)

## Abbreviations

PBRTQC: Patient-based real-time quality control
PBQA: Patient-based quality assurance
IQC: Internal quality control
EQA: External quality assessment
CPP: Clustering-PBRTQC-PBQA
OPTICS: Ordering Points To Identify the Clustering Structure
EWMA: Exponentially weighted moving average
HD50: Harrell-Davis 50th percentile estimator
MD: Mahalanobis distance
WBC: White blood cell count
RBC: Red blood cell count
HGB: Haemoglobin
PLT: Platelet count
MCV: Mean corpuscular volume
LIS: Laboratory information system
HIS: Hospital information system
