# Supplementary material for: Clustering of patient-derived laboratory data: A privacy-preserving distributed approach to enhance clinical laboratory quality control and regional external quality assessment
Source: PLoS One. 2026 Oct 16;21(10):e0359083. doi: 10.1371/journal.pone.0359083 (PMC13674710; doi:10.1371/journal.pone.0359083)
Supplement: Supplementary file 1 [file pone.0359083.s002.docx]

**S1 Table. Head-to-head comparison of alert episodes: Conventional PBRTQC vs. Clustering-PBRTQC-PBQA(CPP)**

**Part A. Overall alert episodes by Westgard rule (all analytes, all seven hospitals)**

| **Alert Rule** | **Conventional PBRTQC** | **CPP** | **Change** | **Change (%)** |
| --- | --- | --- | --- | --- |
| EWMA | 92 | 69 | -23 | -25.0% |
| 1-3S | 64 | 84 | +20 | +31.2% |
| 2-2S | 24 | 20 | -4 | -16.7% |
| **Total** | **180** | **173** | **-7** | **-3.9%** |

**Part B. Per-analyte EWMA alert episode changes**

| **Analyte** | **EWMA Change (%)** | **Interpretation** |
| --- | --- | --- |
| WBC | -100% | Complete elimination of systematic bias signals |
| PLT | -72.2% | Substantial reduction; high biological variability analyte benefits most from population filtering |
| MCV | +21.1% | Low-variability analyte; modest increase reflects preserved sensitivity to random errors |
| RBC | Minimal change | Stable analyte with low biological variability |
| HGB | Minimal change | Moderate variability; selective filtering effect |
